# Intraoperative Dissociation and Migration of the Trial Bipolar Cup in Hip Hemiarthroplasty: A Rare Case

**DOI:** 10.7759/cureus.62180

**Published:** 2024-06-11

**Authors:** Danai Grammatikopoulou, Christina Pechlivani, Konstantinos Asteriadis, Aristeidis Vrettakos, Georgios Antonoglou

**Affiliations:** 1 Department of Orthopedic Surgery and Traumatology - Unit for Sport Injuries, General Hospital of Thessaloniki “Agios Pavlos”, Thessaloniki, GRC; 2 Department of Anatomy-Histology-Embryology, University of Ioannina, Ioannina, GRC

**Keywords:** intraoperative complication, bipolar hemiarthroplasty, trial head dissociation, femoral head trial loss, implant failure, trial head migration

## Abstract

Femoral neck fractures are an ever-increasing pathology, and with the elderly population on the rise, cases of cemented bipolar hemiarthroplasties are also on the rise. This is a rare case of intraoperative dissociation and migration of the trial components of bipolar hemiarthroplasty. Considering the current literature, all junior surgeons should be aware of this possible development during trial reduction. We present the case of an 82-year-old Caucasian woman suffering from a left femoral neck fracture due to a fall. She was treated surgically with a cemented bipolar hemiarthroplasty, but after trial reduction, the trial components dissociated and migrated inside the pelvis. The attempts at recovery through the current approach failed, and a new incision and approach were needed. A small ilioinguinal incision was performed, and the recovery of the trial cup was successful. The patient recovered with no considerable problems. As the reasons for this rare complication are largely unknown, the surgeon should be careful and take measures to prevent this scenario. Moreover, it is wise to weigh the pros and cons of retrieval through other approaches and choose the best course of action for the patient.

## Introduction

The aging of the population has brought about a spike in the incidence of femoral neck fractures, as well as their complications and the high mortality rate that accompanies them [1]. Many protocols have been suggested, and the best way to treat patients with displaced femoral neck fractures is still under debate. However, there is unanimity that elderly patients over the age of 75 years with a plethora of comorbidities are better treated with cemented bipolar hemiarthroplasty as soon as possible due to shorter hospital stays, shorter operating durations, and lower revision rates [1].

In this case report, an intraoperative complication is presented, whereas during trial reduction and dislocation, the trial head migrated away from the hip, and a second incision was needed for its retrieval. Although very rare in bipolar hemiarthroplasty [2], a few similar cases have been reported during total hip arthroplasty. In this case, the patient had a full recovery with no major complications, but that is not always the case [2,3]. Intraoperative dissociation and migration of trial components in bipolar hemiarthroplasty, though rare, is a recognized complication that can lead to significant clinical challenges. The etiology of this phenomenon is unknown, although poor technique, faulty trial prostheses, and unique patient anatomy could be at fault.

## Case presentation

History

An 82-year-old Caucasian woman was transferred to our emergency department due to a fall and an inability to bear weight on her left lower limb. Before the fall, the patient was ambulatory. From her history, the patient suffered from high blood pressure, hyperlipidemia, type II diabetes mellitus, paroxysmal atrial fibrillation, chronic renal failure, hypothyroidism, and coronary artery disease. She had undergone coronary angioplasty and stent placement in two arteries, as well as a pacemaker installation, 15 years before the fall, with no other history of surgical intervention. Her conditions were adequately maintained with medication.

Signs and symptoms

The patient complained about pain in the left hip joint and an inability to sit or ambulate. Her left lower limb was shortened in external rotation and abduction. She had a positive Patrick’s test with a limited range of movement of the left hip, reproducing the patient’s pain. There was no sign of perfusion failure or altered neurological status of the left lower limb.

Radiographic evaluation

**Figure 1:**
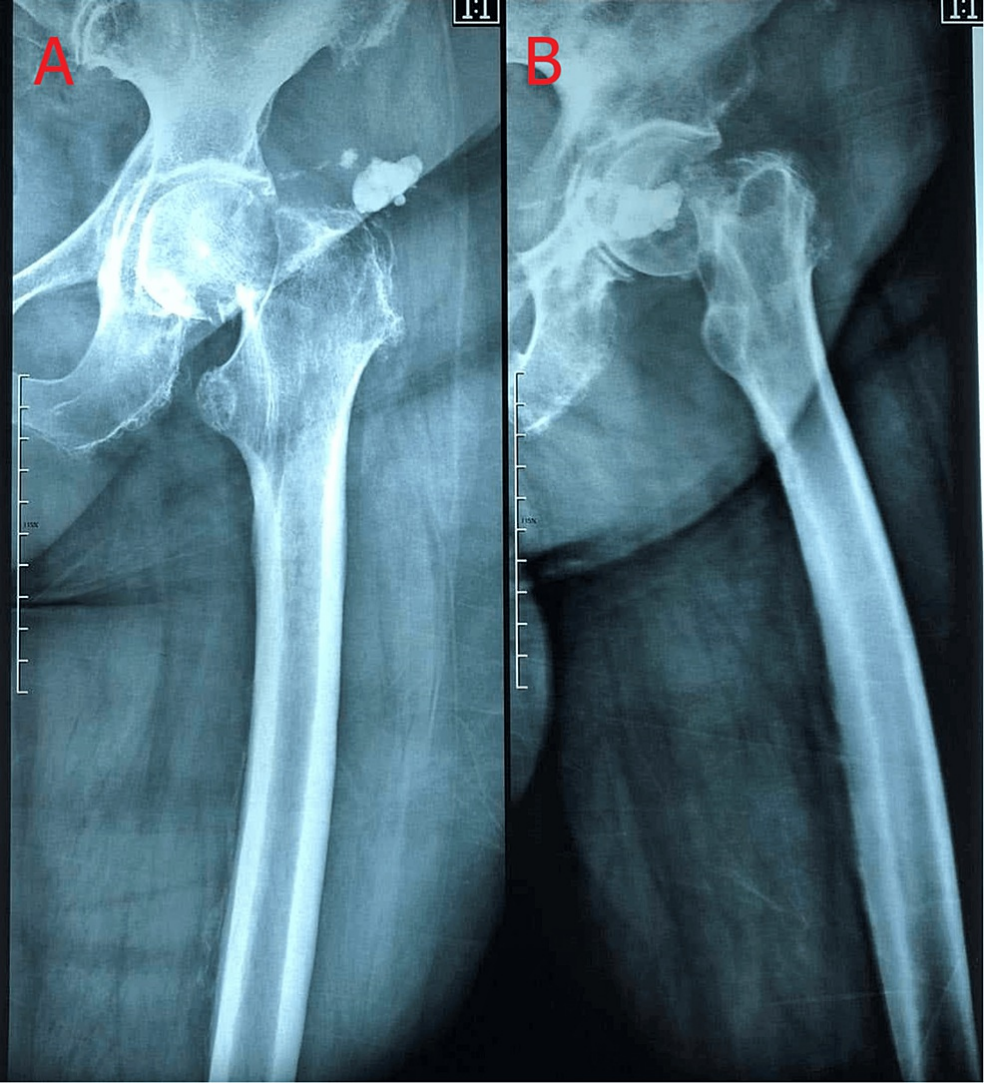
Left hip radiographs. A: Anteroposterior left hip radiograph. B: Profile left hip radiograph. These radiographs were taken when the patient first arrived at the emergency department.

Chest, left hip, pelvis, and lumbar spine anteroposterior radiographs, as well as profile left hip and lumbar spine radiographs, were performed that showcased the femoral neck fracture of the left hip (Figure 1).

Treatment

**Figure 2:**
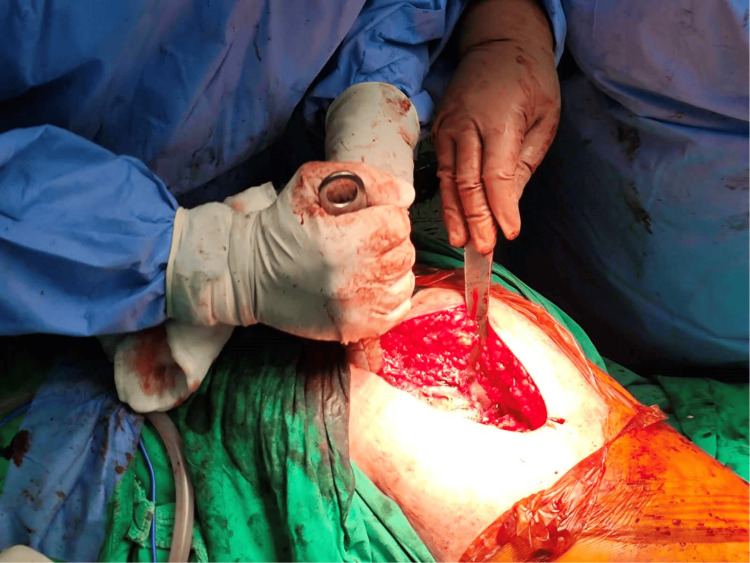
Lateral approach of the left hip. Intraoperative image after a direct lateral incision and a modified Hardinge’s approach of the left hip were performed.

**Figure 3:**
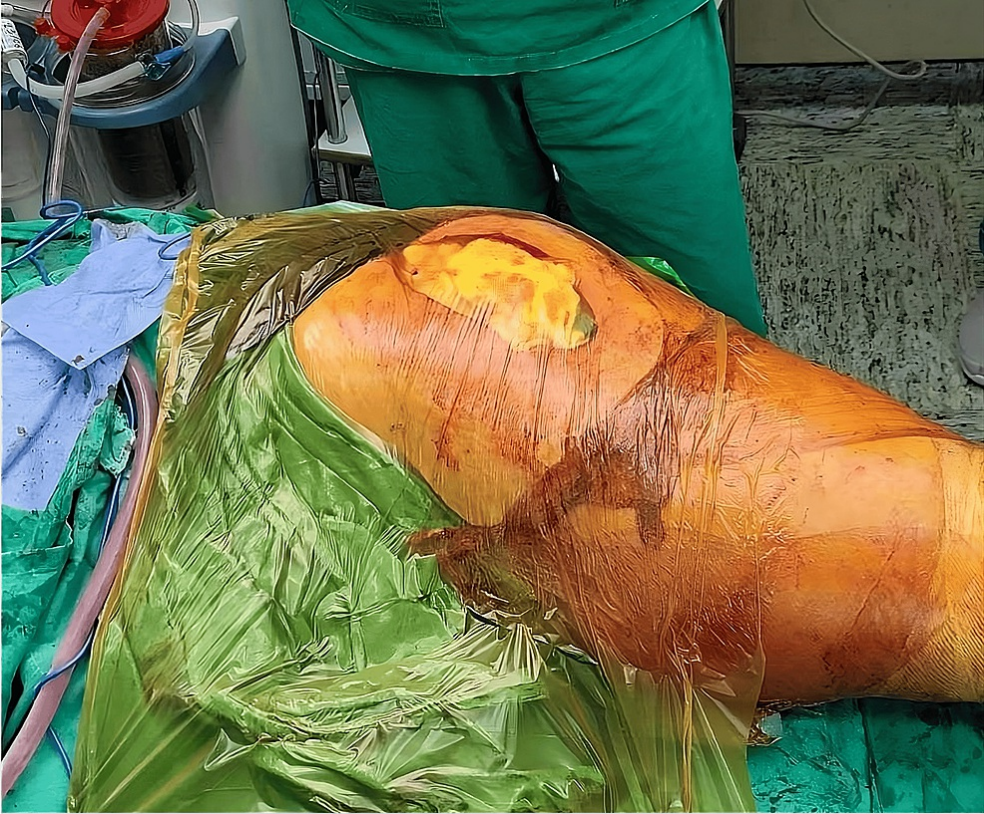
Preparation and sterilization of the operative field anew. The operative field was sterilized again before the patient was repositioned in a supine position.

**Figure 4:**
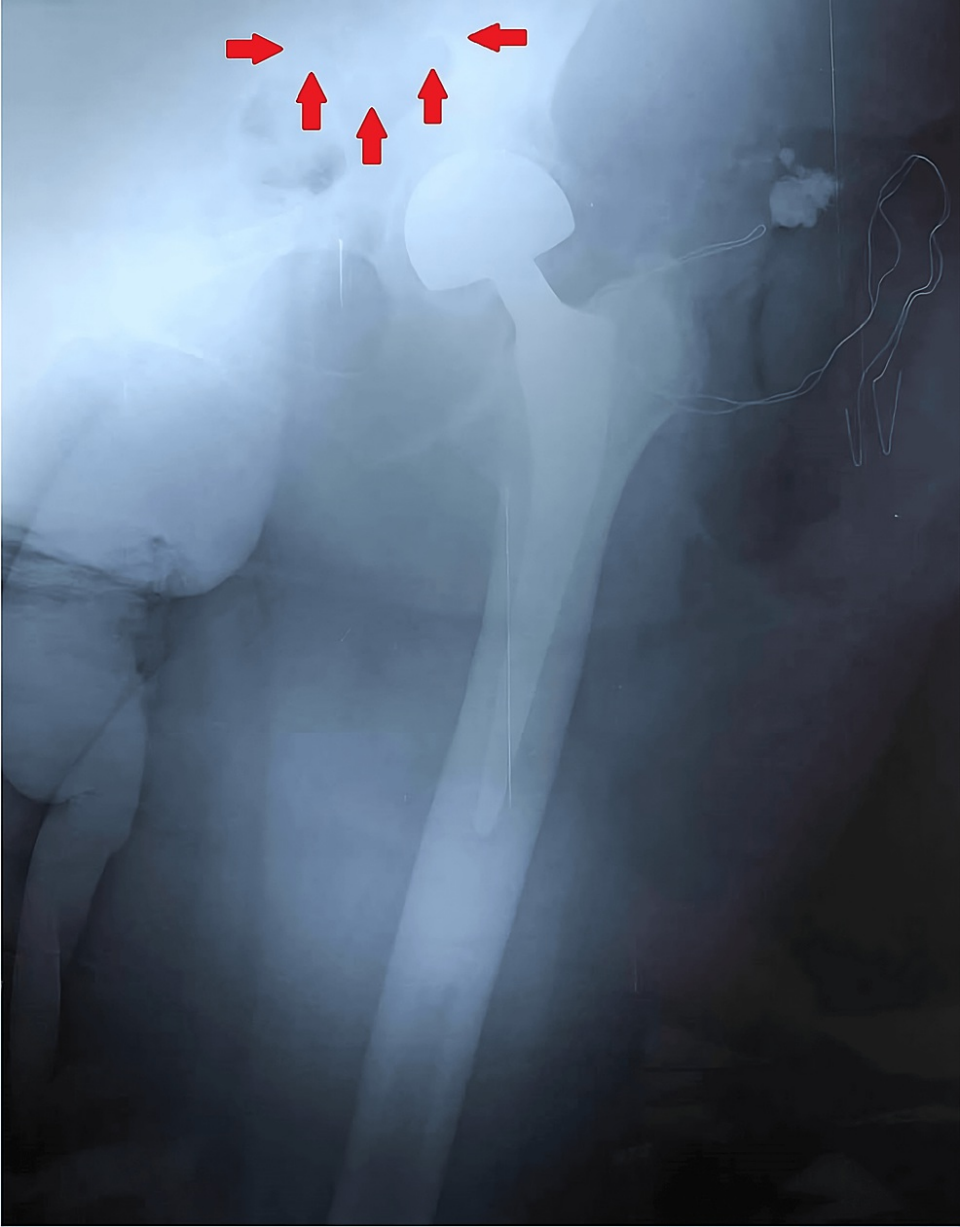
Intraoperative left hip radiograph. Left hip anteroposterior radiograph, after supine positioning of the patient, to facilitate the detection of the trial components, with no success due to their inherent radiolucency. The arrows mark the area where trial components were eventually retrieved (between the psoas muscle and the transverse abdominal muscle).

**Figure 5:**
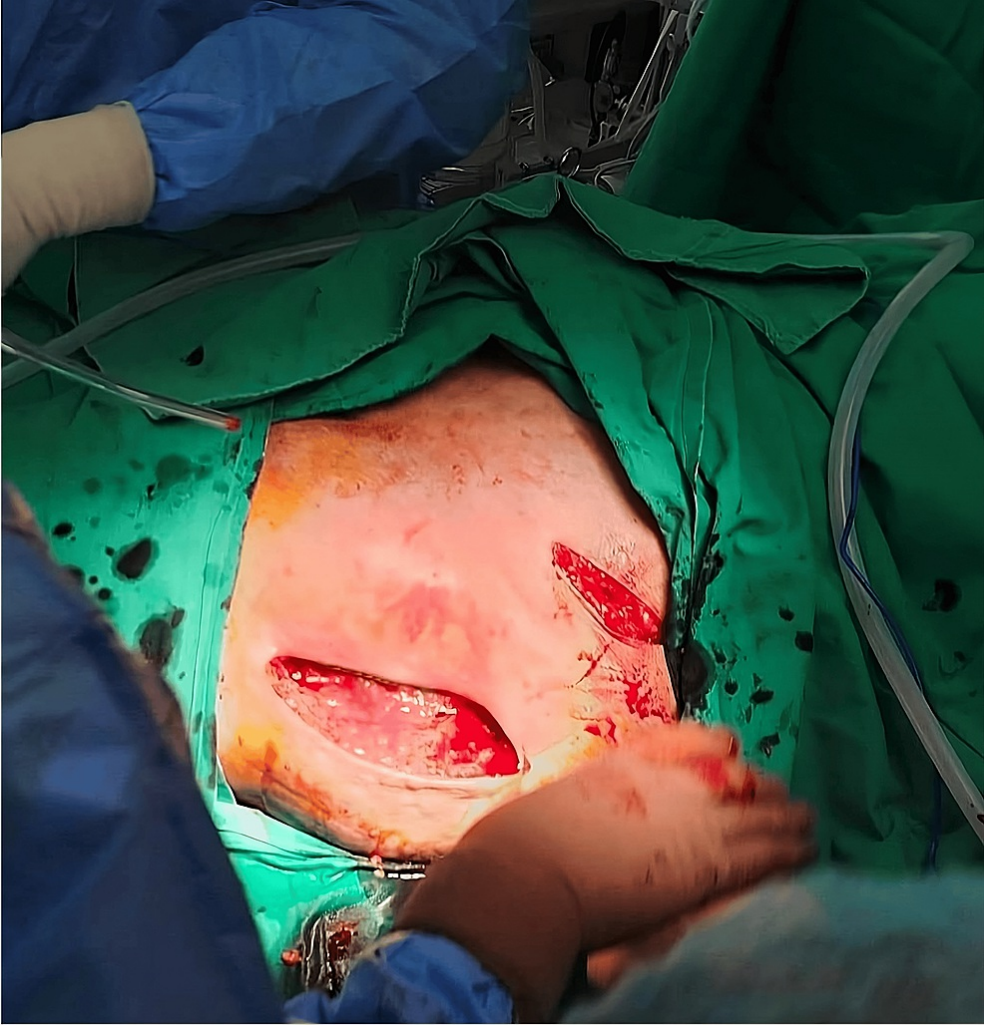
Second incision performed to retrieve trial components. A minimal ilioinguinal approach was performed, in which the trial components were retrieved easily from between the psoas and the transverse abdominal muscle.

**Figure 6:**
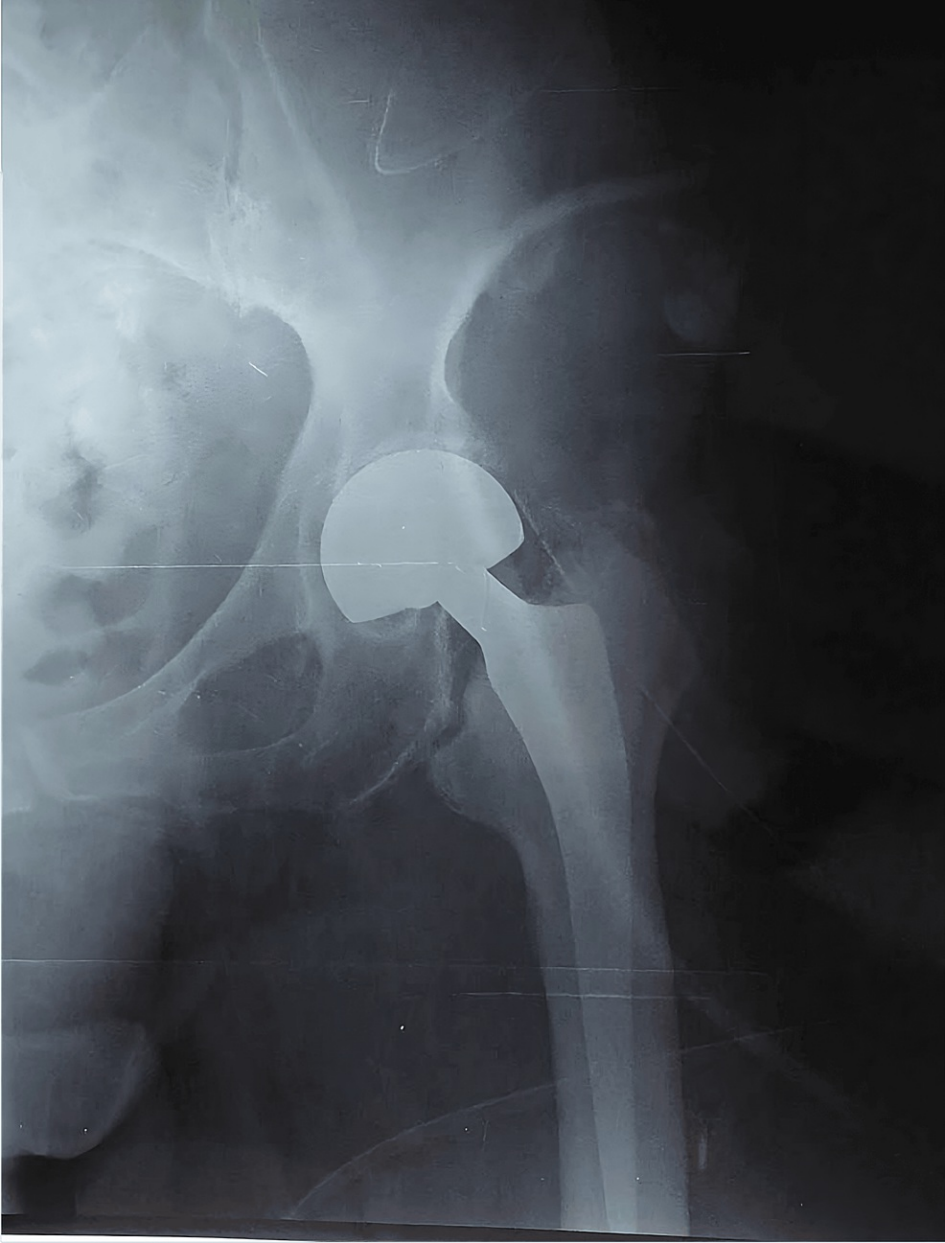
Postoperative left hip radiograph. Good positioning of the left hip hemiarthroplasty components was confirmed.

Due to the use of dabigatran by the patient, the surgery was performed three days after admission. The patient was placed in the lateral decubitus position under spinal anesthesia. A direct lateral incision and a modified Hardinge’s approach (Figure 2) were performed by a first-year fellow under the supervision of a senior consultant. After the removal of the femoral head and the broaching of the femur with a size 11 broach, the trial reduction was attempted with a trial cup measuring 45 mm and a short trial femoral neck. The trial reduction was successful but slightly more arduous than expected. During the dislocation of trial components, both the trial cup and neck were dissociated from the femoral broach, turned, and migrated far into the anterosuperior area of the hip. All endeavors to be grasped with fingers, forceps, and graspers failed, with some even exacerbating the migration. Eventually, the continuation of the cemented bipolar hemiarthroplasty was decided, and the final components were implanted successfully. The patient was then repositioned in a supine position, and fluoroscopy was employed to facilitate the detection of the trial components, with no success due to their inherent radiolucency (Figures 3, 4). Additional imaging evaluation, such as ultrasound, was not applied intraoperatively to detect the trial components. Though an efficient technique, it was decided that the examination would alter the operative time further. In the case that a laparotomy was needed, a general surgeon was scrubbed in. Finally, a minimal ilioinguinal approach was performed, in which the trial components were easily retrieved from between the psoas muscle and the transverse abdominal muscle (Figure 5). Subsequently, both incisions underwent a rigorous lavage and were closed with drainages installed, which were removed on the second postoperative day (Figure 6).

Postoperative protocol

The patient suffered minor complications after the surgery, mainly a low-grade fever on the first postoperative day, delirium, and anemia, that required blood transfusion on the second postoperative day, and, eventually, she was discharged after a week. She managed to ambulate during her stay at a rehabilitation center 13 days after her surgery. During the follow-up check after three months, she was walking without an aid.

## Discussion

There are several reported cases of postoperative disengagement of the components of hemiarthroplasty or total hip arthroplasty [3], but disengagement of the trial components is a rarer scenario [4]. This dissociation occurs during reduction attempts, stability assessment, or dislocation after the trial. All hip approaches have an incidence of this complication, with the minimally invasive techniques having a higher one, possibly because of the small and tight operative field that facilitates migration and hinders maneuverability [4].

Several theories have been proposed regarding why this migration occurs. Whereas some of these concern the patient, such as muscle atrophy, weight loss, or obesity [4,5], a multitude of them are related to the surgical technique. In particular, minimally invasive approaches, extensive anterior capsulotomy, instability, impingement, not checking that trial components are fitted appropriately, or inexperience in performing the above may result in dissociation of the trial components [6,7]. Lastly, some theories support the idea that the component design is at fault or that repeated sterilization wears down the material enough that, at some point, disengagement at the trial stage is inevitable [4].

The most common location of migration is anterior to the rim of the acetabulum, under the inguinal canal, and following the anterior of the psoas muscle. The component is trapped in the retroperitoneum, usually below the anterior third of the iliac crest [8]. Rarer locations that have been described include the sciatic notch [9] or the area behind the pubic ramus [5].

Many surgeons who reported cases of intraoperative migration of the trial head in total hip arthroplasty decided to complete the surgery and assess the patient in the postoperative period for complications, especially when the patient was in poor condition. They theorized that the trial head is smooth, small, and bio-inert enough that more damage would befall the patient in an attempt to track the trial head in the pelvis, especially if its location was unknown due to its radiolucency. In this case study, though, the trial cup was rather large and had some corners, so there was concern for pressure on neurovascular structures. There have been instances in the aforementioned cases where the patients developed abdominal pain, neurovascular complications, hip pain, and even an infection due to the non-removal of trial components. Subsequently, these patients had to be operated on a second time [6,10].

Bicanic et al. [10] developed an algorithm for the management of these cases, while Abouel-Enin et al. [5] summarized all the different techniques that can be used, as well as a plethora of tips and tricks. Moreover, manual attempts using hemostatic forceps with pressure on the hypogastrium or a Satinsky/Debakey clamp for retrieval are not advised [11]. In case the abovementioned procedure fails, a second incision, which can be ilioinguinal, Stoppa, Smith-Peterson, Rutherford-Morrison, or even laparotomy, is employed. Thereafter, the trial component can be retrieved directly or indirectly by pushing through the first incision toward the second.

Some methods to prevent this complication from occurring have been mentioned. Alfonso et al. used a heavy suture passed through the holes of the trial heads to create a “trial head necklace,” making retrieval upon dissociation easier [12]. Others used anterior space packing if there was a need for extensive capsulotomy or if there was a large space near the anterior capsule with poor quality of the iliofemoral and pubofemoral ligaments [6]. Lastly, frequent inspection of the trial components for potential wear should be performed [7].

## Conclusions

In the case presented, the patient’s recovery was not severely altered by the complication of migration, but in other cases, it had a mild to detrimental effect on the patient’s life. Caution must be exercised, especially in elderly patients with muscle atrophy and modified anatomy structures because of osteoporosis, arthritis, or other diseases that can plague the joints and their relative anatomy. Thus, the clinical message of this study is summarized as follows: if the migrating component can cause no severe complications in elderly patients due to its location or physical attributes, then retrieval may not be suitable for these patients, as the duration of the surgery is shortened and any morbidity associated with a second approach is avoided. If retrieval is possible and can be quick and minimally invasive, it is recommended. Nevertheless, the best way to handle this complication is prevention.
